# Drugs to Block Cytokine Signaling for the Prevention and Treatment of Inflammation-Induced Preterm Birth

**DOI:** 10.3389/fimmu.2015.00166

**Published:** 2015-04-20

**Authors:** Pearl Y. Ng, Demelza J. Ireland, Jeffrey A. Keelan

**Affiliations:** ^1^King Edward Memorial Hospital, School of Women’s and Infants’ Health, University of Western Australia, Perth, WA, Australia

**Keywords:** chorioamnionitis, cytokine suppressive anti-inflammatory drugs, intrauterine inflammation, intrauterine infection, NF-κB inhibitors, preterm birth

## Abstract

Preterm birth (PTB) at less than 37 weeks of gestation is the leading cause of neonatal morbidity and mortality. Intrauterine infection (IUI) due to microbial invasion of the amniotic cavity is the leading cause of early PTB (<32 weeks). Commensal genital tract *Ureaplasma* and *Mycoplasma* species, as well as Gram-positive and Gram-negative bacteria, have been associated with IUI-induced PTB. Bacterial activation of Toll-like receptors and other pattern recognition receptors initiates a cascade of inflammatory signaling via the NF-κB and p38 mitogen-activated protein kinase (MAPK) signaling pathways, prematurely activating parturition. Antenatal antibiotic treatment has had limited success in preventing PTB or fetal inflammation. Administration of anti-inflammatory drugs with antibiotics could be a viable therapeutic option to prevent PTB and fetal complications in women at risk of IUI and inflammation. In this mini-review, we will discuss the potential for anti-inflammatory drugs in obstetric care, focusing on the class of drugs termed “cytokine suppressive anti-inflammatory drugs” or CSAIDs. These inhibitors work by specifically targeting the NF-κB and p38 MAPK inflammatory signaling pathways. Several CSAIDs are discussed, together with clinical and toxicological considerations associated with the administration of anti-inflammatory agents in pregnancy.

## Introduction

Preterm birth (PTB), delivery prior to 37 weeks of gestation, is estimated to affect 5–15% of pregnancies worldwide (1) and remains the leading cause of morbidity and mortality of neonates (2) and the second largest direct cause of death in children under 5 years (3). There are many pathological pathways which can lead to PTB, including intrauterine infection (IUI), uterine ischemia, uterine over-distension, abnormal allogeneic recognition and allergic reactions, cervical disease, and endocrine disorders (4). Whilst interventions such as progesterone therapy and ultrasound cervical monitoring are now utilized in many high-risk clinics in developed countries, they are not specific to a particular PTB etiology (5). IUI and inflammation has been casually linked to early PTB and account for approximately 40% of all spontaneous PTB (6, 7). Microbial invasion of the amniotic cavity, most commonly with ascending vaginal microorganisms, activates pattern recognition receptors (PRRs) which induce the production of pro-inflammatory mediators leading to the premature activation of labor and ultimately PTB [reviewed in Ref. (8)]. Neonatal health and development is further compromised if chronic exposure of the fetus to these inflammatory mediators results in fetal inflammatory response syndrome (FIRS) (9, 10). It is likely that optimal pregnancy outcomes will come from the development of therapeutic strategies that are cause-specific and targeted to women at risk and likely to benefit from the treatment.

There is growing interest in therapeutic interventions that target the inflammatory labor cascade by blocking the production of pro-inflammatory mediators or up-regulating anti-inflammatory mediators and/or the exogenous administration of anti-inflammatory or pro-resolution mediators. In their review of anti-inflammatory agents for the prevention of labor, Rinaldi et al. (8) concluded that progesterone was the most likely compound to progress into mainstream clinical use. Although progesterone treatment reduces the incidence of preterm delivery (11), its ability to block inflammatory signaling associated with infection is unclear. In a previous review, we concluded that while the use of anti-inflammatory agents for the treatment and/or prevention of PTB appears promising, pre-clinical studies demonstrating clear benefits and lack of toxicity are needed (12). This mini-review will revisit the benefits and risks of administration of anti-inflammatory drugs in obstetric care, focusing specifically on the emerging class of drugs termed “cytokine suppressive anti-inflammatory drugs” or CSAIDs. These inhibitors, when administered in conjunction with an effective antibiotic regimen, have the potential to both prolong pregnancy and improve neonatal outcomes.

## Intrauterine Infection and Inflammation

One in four preterm infants is born to a mother with IUI (13). These infections commonly arise via the ascending route of infection where bacteria from the cervicovaginal fluid ascend, breach the cervical barrier, colonize the amniotic fluid, and invade fetal membranes and in some cases the fetus (14). IUI also occurs via hematogenous dissemination with trans-placental transfer (15). Chorioamnionitis is the hallmark feature of IUI and is typically diagnosed after delivery by histopathology as the infiltration of leukocytes into the fetal membranes; histological chorioamnionitis severity is positively correlated with intra-amniotic infection, fetal inflammation, and poorer pregnancy outcomes (16, 17). While bacteria are the major organism responsible for chorioamnionitis, viruses and yeast are also capable of causing intrauterine inflammation.

**Figure 1:**
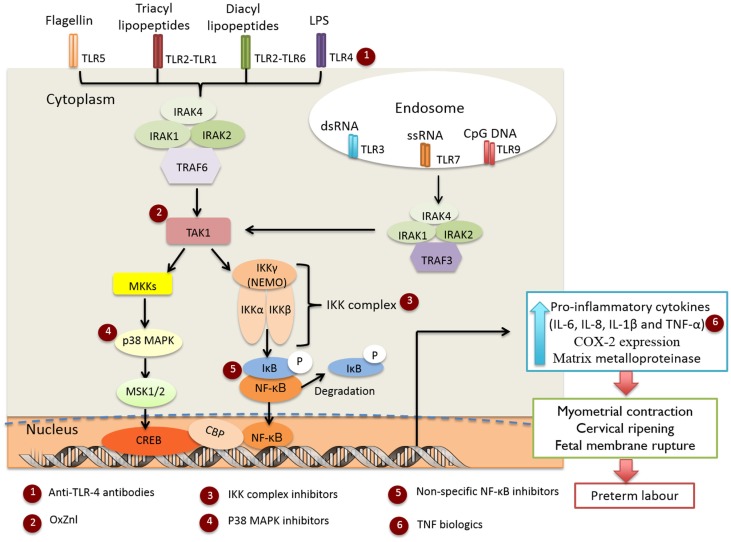
**Infection-induced preterm labor triggered by activation of TLR-mediated NF-κB and p38 MAPK inflammatory signaling cascades**. Targets for the selected anti-inflammatory agents are indicated in red circles.

Genital *Mycoplasma* and *Ureaplasma* species are some of the most commonly isolated organisms from amniotic fluid in cases of infection-induced PTB (7), although the appearance of these, and numerous other bacteria (7, 18), in amniotic fluid does not necessarily denote causation (19). Evidence suggests that the extent of bacterial colonization, route of infection, and the stimulatory capacity of the bacteria all play key roles in the activation of maternal and fetal pro-inflammatory signaling cascades which induce production of pro-inflammatory cytokines (e.g., IL-1β and TNF-α) and chemokines (e.g., IL-8 and MCP-1), which in turn promote prostaglandin (PG) production and myometrial contractility, ripening of the cervix, and degradation of the fetal membrane extracellular matrix leading to preterm labor (PTL) (20). The importance of cytokine and chemokine signaling in the pathogenesis of infection-induced PTL is well established and has been thoroughly reviewed in Ref. (14, 21, 22). Microorganism-specific pathogen-associated molecular patterns (PAMPs) are sensed by trans-membrane PRRs, e.g., Toll-like receptors (TLRs) (23, 24), with ligation resulting in recruitment of adaptor proteins [IL-1R-associated kinase (IRAK)1, IRAK4, and TNF receptor-associated factor (TRAF6)] and activation of TAK1 kinase (Figure 1). TAK1 then mediates the phosphorylation and activation of the IκB kinase complex (IKK), which comprises of two catalytic subunits (IKKβ and IKKα) and a regulatory subunit IKKγ (25). The IKK complex phosphorylates IκB-α, targeting it for degradation, allowing NF-κB heterodimers to dissociate and translocate to the nucleus to drive inflammatory gene expression (26). TAK1 kinase can also phosphorylate and activate the mitogen-activated protein kinases (MAPKs), MKK3 and MKK6 that subsequently activate p38 MAPK (27). Although there is some evidence that p38 MAPK is involved in intrauterine inflammatory activation of fetal membranes (28), the exact mechanism of activation in gestational tissues and pregnancy is unknown and likely varies according to the nature of the stimulatory agent.

## Targeting Pro-Inflammatory Signaling for Prevention or Treatment of PTB

Antibiotic treatment is routinely given to women presenting with PTL (29, 30). However, it is not the infection but the subsequent inflammation that initiates PTL and is primarily responsible for adverse neonatal outcomes. The use of non-steroidal anti-inflammatory drugs (NSAIDs) to inhibit PG synthesis provided initial evidence that the use of anti-inflammatory drugs may help to delay PTB (31, 32). However, significant pregnancy complications and adverse fetal side effects have been associated with their use (33) as summarized in Table S1 in Supplementary Material. The following sections consider a number of promising alternative anti-inflammatory agents with potential for use in preventing inflammation-driven PTB.

### Non-specific NF-κB inhibitors

*N*-acetyl cysteine (NAC) is a non-specific free radical scavenger and NF-κB inhibitor (34–36) (Figure 1, indicated by red circle at position 5). Treatment of fetal membranes with NAC has been shown to inhibit lipopolysaccharide (LPS) and γ-irradiation killed *E. coli*-induced inflammation (37, 38). NAC has been tested clinically in pregnancy, but has not progressed into mainstream clinical use (39) and the clinical findings have not yet been replicated in other studies. Sulfasalazine (SSZ), a salicylate drug that blocks NF-κB activation by directly inhibiting the IKK kinases (40), is well tolerated and approved for use in pregnancy, with no discernible increase in risk of fetal congenital defects, morbidity, or mortality (41). SSZ treatment has been shown to reduce both LPS-induced placental inflammation in an explant model (42), and the incidence of preterm delivery in a mouse model of PTL (43). However, increased levels of chorionic apoptosis has also been reported in a human membrane model after SSZ exposure (20 h), suggesting that prolonged treatment may result in eventual membrane degradation and loss of function and structural integrity (42).

### TLR4 antagonists

TLR4 activation by LPS is the most commonly used IUI model and accordingly TLR4 antagonism has been assessed for therapeutic potential (Figure 1, red circle at position 1). Studies of TLR4–LPS inhibition using a monoclonal anti-TLR4 antibody found treatment effective *in vivo* in reducing pro-inflammatory mediator (TNF-α, IL-8, and PGE_2_) production in amniotic fluid (44), and the incidence of LPS-induced PTB (45). Alternate TLR4 antagonists include eritoran tetrasodium (46) and TAK-242 (47), neither of which have been examined in this context. IUI and inflammation can be triggered by a range of PAMPs, while TLR4 antagonism is only appropriate in cases of Gram-negative bacteria-induced PTL.

### TNF-α biologics

Conflicting reports exist regarding the efficacy of anti-TNF-α antibodies to decrease the incidence of PTB in murine models (48, 49). Drugs blocking the production of pro-inflammatory TNF-α are used in pregnancy (50, 51), but the complexity of cytokine interactions associated with PTL suggests that targeting individual cytokines may not be the most optimal therapeutic intervention (Figure 1, red circle at position 6). Interestingly, clinical studies have reported that maternal administration of antibody-based TNF-α biologics (e.g., infliximab) persist in the neonatal circulation for many weeks after birth (52) and may therefore dampen both intrauterine and fetal inflammation protecting the fetus from the adverse sequelae of IUI and inflammation. There is little evidence for congenital abnormalities with the use of anti-TNF-α therapy during pregnancy (53), but high levels in fetal circulation may increase risk of neonatal infection. The consequences of such treatments for the developing immune system need to be fully considered.

### CSAIDs: A novel class of anti-inflammatory drugs

As a class of compounds, CSAIDs specifically target the NF-κB and p38 MAPK signaling pathways to inhibit cytokine-mediated events with demonstrated efficacy in a range of animal models (54–56). These agents are now being examined for their potential to be more effective and selective than NSAIDs for the inhibition of inflammation-driven PTB, as they directly target signaling molecules leading to the activation of the NF-κB and p38 MAPK inflammatory cascades without interfering with the constitutive/homeostatic roles of prostanoids (Table 1 and Figure 1). Importantly, depending on the route of administration and placental transfer properties, CSAIDs may have the potential to block intra-amniotic and fetal inflammation, thereby protecting the fetus from the adverse sequelae of exposure to inflammatory mediators.

**Table 1:** **Cytokine suppressive anti-inflammatory drugs (CSAIDs) with potential for the prevention or treatment of PTB**.

CSAID	Formula (molecular weight, kDa)	Mode of action	Anti-inflammatory effects in IUI and other models of inflammation	Potential side effects
SKF-86002	C_16_H_12_FN_3_S (297.4 kDa)	Inhibits p38 MAPK, COX-2, and 5-LO enzymes (57)	↓ IL-1β and PGE_2_ production by LPS-stimulated human fetal membranes (58), ↓ IL-1β from endotoxin-stimulated human macrophages (58)	Downstream MAPK inhibitory effects on placental growth and differentiation (59, 60)
SB202190	C_20_H_14_FN_3_O (331.3 kDa)	Selectively binds to the ATP-binding pocket of p38α and β isoforms (61)	↓ IL-6, TNF-α, PGE_2_, and PGE_2α_ production by LPS-stimulated human fetal membranes (28), ↓ IL-6, IL-8, PGE_2_, and PGF2α secretion in macrophage-exposed annulus fibrosis, cells in response to TNF-α (62)	Downstream MAPK inhibitory effects on placental growth and differentiation (59, 60)
SB239063	C_20_H_21_N_4_O_2_F (368.4 kDa)	Selectively binds to the ATP-binding pocket of p38α and β isoforms (61)	↓ IL-6, TNF-α, and PGE_2_ production by γ-irradiation-killed *E. coli* stimulated human fetal membrane Transwell model (38)	Downstream MAPK inhibitory effects on placental growth and differentiation (59, 60)
NBDI	Synthetic peptide corresponding to the NEMO amino-terminal alpha-helical region (3780.4 kDa)	Binds to IKKβ NEMO-binding domain and inhibits kinase activity by disrupting the interaction of IKKβ with NEMO (63)	↓ PGE_2_ production in LPS and *Ureaplasma parvum*-stimulated ovine gestational membrane Transwell model (38), ↓ IL-6, TNF-α, and IL-1β production and expression in a dose-dependent manner in a mouse model of inflammatory bowel disease (63)	Inhibition of NF-κB constitutive activity resulting in non-specific toxicity (64)
Parthenolide	C_15_H_20_O_3_ (248.3 kDa)	Binds to IKKβ and inhibits kinase activity by covalent modification of the cysteine 179 reside in the kinase activation loop (65)	↓ Inflammatory gene expression and production in primary choriodecidual cells (66), ↓ LPS-induced IL-6 and TNF-α in a mouse model (67), ↓ TNF-α and COX-2 expression in TNF-α-stimulated human urothelial cells (68)	Inhibition of NF-κB constitutive activity resulting in non-specific toxicity (64)
TPCA-1	C_12_H_10_FN_3_O_2_S (279.9 kDa)	Selectively binds to the ATP pocket of the IKKβ kinase (69)	↓ Inflammatory gene expression and production in primary choriodecidual cells (66), ↓ TNF-α and PGE_2_ in fetal side of human fetal membrane Transwell model (38), ↓ IL-8 and PGE_2_ LPS ovine gestational membrane explant model (38), ↓ PGE_2_ in a 2-day LPS ovine pregnancy model (70), ↓ IL-1β-induced MMPs expression and NF-κB nuclear translocation in corneal fibroblasts (71), ↓ IL-1β, IL-6, TNF-α, and IFN-γ in mouse model of collagen arthritis (69)	Inhibition of NF-κB constitutive activity resulting in non-specific toxicity (64)
OxZnl	C_19_H_22_O_7_ (362.4 kDa)	Selectively binds to the ATP pocket of TAK1 kinase (72)	↓ PGE_2_ production by LPS-stimulated ovine model of pregnancy (70), ↓ COX-2 production in mouse model of picryl chloride-induced ear swelling (72), ↓ IL-1-induced COX-2 production in mouse embryonic fibroblast (72), ↓ CD40L-induced IL-6, MCP-1, and ICAM1 in mouse model of vascular injury (73)	Downstream inhibitory effects on MAPK activity involved in cell differentiation and apoptosis (74)

*OxZnl, 5z-7-oxozeaenol; ATP, adenosine triphosphate; COX, cyclooxygenase; CSAID, cytokine suppressive anti-inflammatory drug; IFN, interferon; IL, interleukin; IUI, intrauterine infection; IKK, IκB kinase; LPS, lipopolysaccharide; MMP, matrix metalloproteinase; MAPK, mitogen-activated protein kinase; MCP, monocyte chemoattractant protein; NBDI, NEMO-binding domain inhibitor; NEMO, NF-κB essential modulator; NF-κB, nuclear factor κB; PG, prostaglandin; TAK, TGFβ-activated kinase; TNF, tumor necrosis factor*.

#### p38 MAPK inhibitors

The first p38MAPK inhibitor investigated in human extraplacental membranes was SKF-86002, a potent inhibitor of p38 MAPK and less potent inhibitor of cyclooxygenase-2 (COX-2) and 5-lipoxygenase activity (75). This led to research into the use of similar inhibitors, which selectively bind to the adenosine triphosphate (ATP) site of p38 MAPK, to block the placental production of pro-inflammatory cytokines (Figure 1, red circle at position 4). Lappas et al. (28) reported that treatment of LPS-stimulated human fetal membranes with SB202190 inhibited the release of IL-6, TNF-α, and PGs, whilst we demonstrated that SB239063 inhibited the production of IL-6, TNF-α, and PGE_2_ at both the maternal and fetal faces of human fetal membranes stimulated with γ-irradiation-killed *E. coli* (38). This suggested that p38 MAPK may be a useful pharmacological target for prevention of PTL; however, caution is warranted as MAPKs are also involved in many aspects of cell function and signaling, including placental growth and differentiation (59, 60).

#### IKK complex inhibitors

A short, membrane-permeable NEMO-binding domain inhibitor (NBDI) peptide that spans the IKKβ NEMO-binding domain disrupting interaction between NEMO and IKKβ (76) (Figure 1, red circle at position 3), is effective in ameliorating inflammatory responses in ear swelling (77) and colitis (63) mouse models. Recently, NBDI was also shown to inhibit LPS and *Ureaplasma parvum*-induced PGE_2_ production in ovine gestational membranes (38) but not γ-irradiation-killed *E. coli*-induced pro-inflammatory responses in *ex vivo* human fetal membranes (38); differences in binding affinity or endogenous protease activity in human fetal membranes may explain differential efficacy observed.

TPCA-1 (69) and parthenolide (65) are specific IKKβ inhibitors (Figure 1, red circle at position 3) shown to inhibit LPS-induced inflammation and NF-κB nuclear translocation in primary choriodecidual cells (66). Unlike SSZ, no impairment of cell viability, apoptosis, or expression of anti-apoptotic genes was detected in these studies (66). Addition of TPCA-1 to the fetal compartment of a human fetal membrane Transwell model was also reported to inhibit γ-irradiation-killed *E. coli*-induced TNF-α and PGE_2_ production in the fetal compartment, and to a lesser extent in the maternal compartment (38). TPCA-1 also blocked LPS- and *U. parvum*-induced IL-8 and PGE_2_ production in an ovine gestational membrane model (38), demonstrating that this pharmacological strategy is likely to work across a wide range of microbial stimuli and different PAMPs. Significantly, in an ovine model of LPS-induced chorioamnionitis, intra-amniotic administration of TPCA-1 was found to inhibit production and accumulation of PGE_2_ in amniotic fluid and leukocytosis of the fetal membranes (70). However, at the doses employed, no significant changes in amniotic fluid or fetal circulating cytokine concentrations were observed.

#### TAK1 inhibitors

The TAK1 kinase complex is unique to the TLR-mediated activation pathway and offers an excellent pharmacological target within the p38 MAPK and NF-κB pro-inflammatory signaling cascades to block premature activation of labor, without the downstream effects of IKK inhibition on constitutive NF-κB activity (Figure 1, red circle at position 2). The upstream location of TAK1 also suggests that blockade of activity is likely to exert broad-spectrum anti-inflammatory effects against the range of microbes and stimuli associated with IUI and inflammation. Gene deletion of TAK1 impairs IKK and NF-κB activity, subsequently blocking pro-inflammatory cytokine release and expression (78). To date, availability of pharmacological TAK1 inhibitors is extremely limited; 5z-7-oxozeaenol (OxZnl), a resorcyclic acid lactone and selective inhibitor of TAK1 kinase, appears to be the most promising (79) and has been shown to inhibit pro-inflammatory mediator production in murine fibroblasts (72), primary cortical neurons (80), dermal fibroblasts (81), and ear swelling models (72). In an ovine model of LPS-induced chorioamnionitis, we recently demonstrated intra-amniotic treatment with OxZnl to reduce amniotic fluid levels of PGE_2_ and fetal membrane leukocyte infiltration (70), although intra-amniotic cytokine levels were not altered. Whilst these studies suggest that TAK1 kinase inhibitors might be an effective approach to prevent inflammation-induced PTL, caution is warranted as TAK1 is also involved in MAPK signaling which regulates cell function and signaling, including apoptosis and differentiation (74). Further studies are required to fully define the role of TAK1 in pregnancy and the clinical potential of TAK1 inhibitors.

## Considerations for the Clinical Translation of CSAIDs

### Mode of drug delivery: Sides effects and efficacy

In the context of PTB prevention, therapeutics should ideally eliminate the microorganism from the amniotic cavity, block the ensuing cytokine cascade that drives release of PGs and matrix metalloproteinases (MMPs), prevent the onset of PTL, and minimize risk of FIRS. While prophylactic antibiotic trials have not been overly encouraging, there have been some successes (82) and exciting new antibiotics such as solithromycin hold great promise (83). However, therapies that deal solely with the infection, without suppressing inflammation, are unlikely to achieve maximal benefit. The NAC clinical trial of Shahin et al. (39) demonstrated that maternal administration of CSAIDs (following antibiotics to treat bacterial vaginosis) could prevent PTB and improve neonatal outcomes, although it should be noted that the concentrations of NAC achieved in amniotic fluid and fetal blood following maternal administration were not determined.

Given the wide range of genes controlled by NF-κB, the inhibition of NF-κB activation by CSAIDs could have unwanted side effects. Maternal administration may inhibit NF-κB-dependent innate immune defenses, increasing susceptibility to infections (84). Caution is also warranted regarding the possibility of non-specific fetal toxicity. Observations that p38 MAPK null mice are non-viable (57) highlight the need to investigate the safety and toxicity of p38 MAPK inhibitors during pregnancy. While there are no published studies on the teratogenic effects of IKK inhibitors, complete inhibition of NF-κB activation by IKKβ gene deletion (IKKβ^−/−^) resulted in embryonically lethal uncontrolled apoptosis in the liver of mice (64). Heterozygous IKKβ^+∕−^ embryos developed with normal livers, despite approximately 50% reduction in IKKβ activity, suggesting that modest inhibition in the fetus may be tolerated (64). The pharmacodynamic profile of TPCA-1 appears promising, and the lack of toxicity *in vitro* and *in vivo* suggests that this could be a useful therapeutic approach for the treatment of PTL. Intra-amniotic treatment with competitive ATP protein kinase inhibitors TPCA-1 (IKKβ inhibitor) and OxZnl (TAK1 inhibitor) in an ovine model of LPS-induced chorioamnionitis showed that the CSAIDs were well tolerated by the fetus, at least in the short-term, with no obvious changes in birth weight or fetal liver function observed (70). This suggests that modest reduction in the activity of upstream kinases IKKβ and TAK1 is unlikely to result in the complete suppression of NF-κB activity and non-specific toxicity. Clearly, such concerns are drug- and dose-dependent, requiring extensive and longer-term safety studies before clinical introduction.

Alternatively, it is possible to deliver anti-inflammatory agents directly to the amniotic cavity via ultrasound guided intra-amniotic injection. This route will likely enhance efficacy by delivering the minimal effective dose to target tissues and minimizing unintended exposures and side effects. Depending on the compound, delayed clearance from the amniotic cavity may in fact enhance efficacy and allow single-dose therapy. Recently, we reported that the anti-inflammatory effects of TPCA-1 and SB239063 administered to the amniotic face in a human fetal membrane model were primarily restricted to the fetal compartment, suggesting a lack of trans-membrane transfer (38). The potential benefits of amniotic drug delivery must always be counterbalanced by an assessment of the risks. The procedure-associated risk of spontaneous miscarriage following second trimester amniocentesis is low, with a recent large study finding a non-significant 0.6% increase in miscarriage compared to controls over a 15-year period (85). How this compares to the risks of a third trimester intra-amniotic injection is not known, although the risks at later gestations are likely to be lower than at 12–20 weeks. Nevertheless, it would be prudent that intra-amniotic treatment be given selectively to women in whom a significant benefit from CSAID therapy can be expected.

### Identification of women at risk: Short cervix and inflammation

Intrauterine infection is often chronic and usually asymptomatic until the presentation of PTL, at which time it is often too late to treat and the fetus has been irreversibly exposed (86). The early identification of women at high risk of adverse pregnancy outcome associated with IUI, before the presentation of clinical symptoms, is challenging but also key for the successful prevention of PTB and improvement of neonatal outcomes. Analysis of amniotic fluid/cervicovaginal fluid cytokine levels or microbial status have been explored to identify women at an elevated risk of PTB (87), but have lacked specificity and/or sensitivity. Sonographic studies have reported that a short cervix (cervical length ≤25 mm) is associated with intra-amniotic inflammation, and patients with this condition are at increased risk of adverse pregnancy outcome (88, 89). Gomez et al. (90) reported that women with a cervical length of ≤15 mm between 22 and 30 weeks of gestation have a higher rate of microbial invasion of the amniotic cavity (43 vs. 3.9%; *p* < 0.05), and were more likely to deliver spontaneously before 35 weeks of gestation (66.7 vs. 13.5%; *p* < 0.01). These studies suggest that assessing sonographic cervical length may be a useful predictor of risk of microbial invasion of the amniotic cavity and intra-amniotic inflammation (89).

## Summary and Conclusion

Infection and inflammation is the leading cause of PTB, but antenatal antibiotic treatment has had limited success at preventing PTB or improving neonatal outcome (30, 91). Newer macrolide antibiotics such as solithromycin, with greater efficacy and better trans-placental passage, may prove in time to be more effective (83, 92). We propose that a combination of anti-inflammatory therapy and effective antibiotics will be required to combat IUI and reduce the associated inflammatory responses leading to PTL and adverse fetal sequelae. Intra-amniotic delivery offers significant advantages in terms of dose reduction, localized site of action, and reduction in potential side effects. CSAIDs, novel compounds that specifically target cytokine signaling pathways, have anti-inflammatory actions in both human fetal membranes *in vitro* and animal models of IUI. These compounds have the potential to be safer and more effective than less selective inhibitors as they target key molecules involved in the pro-inflammatory signaling cascades that prematurely trigger labor. Issues regarding maternal and fetal toxicity, mode of drug delivery, off-target side effects, and appropriate identification of women requiring treatment remain to be addressed. Based on our current appreciation of the importance of IUI and inflammation in the etiology of PTB, the identification and treatment of pregnant women at risk of IUI with effective cytokine signaling inhibitors holds great promise for the prevention of PTB and improvement of neonatal outcomes.

## Conflict of Interest Statement

The authors declare that the research was conducted in the absence of any commercial or financial relationships that could be construed as a potential conflict of interest.

## Supplementary Material

The Supplementary Material for this article can be found online at http://journal.frontiersin.org/article/10.3389/fimmu.2015.00166

## Abbreviations

ATP, adenosine triphosphate; COX, cyclooxygenase; CSAID, cytokine suppressive anti-inflammatory drug; FIRS, fetal inflammatory response syndrome; IκB, inhibitor of NF-κB; IKK, IκB kinase; IL, interleukin; IRAK, IL-1R-associated kinase; IUI, intrauterine infection; LPS, lipopolysaccharide; MAPK, mitogen-activated protein kinase; MCP, monocyte chemoattractant protein; MKK, MAPK kinase; MMP, matrix metalloproteinase; NAC, *N*-acetyl cysteine; NBDI, NEMO binding domain inhibitor; NEMO, NF-κB essential modulator; NF-κB, nuclear factor κB; NSAID, non-steroidal anti-inflammatory drug; OxZnl, 5z-7-oxozeaenol; PAMP, pathogen-associated molecular pattern; PTB, preterm birth; PTL, preterm labor; PG, prostaglandin; SSZ, sulfasalazine; TAK, TGFβ activated kinase; TGF, transforming growth factor; TLR, Toll-like receptor; TNF, tumor necrosis factor; TRAF, TNF receptor-associated factor.
