# Supplementary material for: Drugs to Block Cytokine Signaling for the Prevention and Treatment of Inflammation-Induced Preterm Birth
Source: Front Immunol. 2015 Apr 20;6:166. doi: 10.3389/fimmu.2015.00166 (PMC4403506; doi:10.3389/fimmu.2015.00166)
Supplement: Supplementary file 1 [file Table_1.DOCX]

**Supplementary Table 1: Anti-inflammatory agents for the prevention and treatment of PTB**

| **Anti-inflammatory agents** | **Mechanism** | **Inhibitory effects in IUI and pregnancy models** | **Benefits** | **Potential side effects** |
| --- | --- | --- | --- | --- |
| Non-specific NF-κB inhibitor: NAC | Regulate oxidative stress by increasing glutathione levels and ameliorating inflammation ([91](#_ENREF_91)) | ↓ IL-6, IL-8, TNF-α and PGE_2_ production in LPS stimulated human fetal membranes ([35](#_ENREF_35))  ↓ LPS-induced PTL in a mouse model and attenuate inflammation induced fetal brain injury ([92](#_ENREF_92)) | A clinical study shown a reduction in PTB rates and neonatal mortality when co-administered with 17-hydroxyprogesterone([37](#_ENREF_37)) | Unpleasant to consume with a drop-out rate of 11% ([37](#_ENREF_37)) |
| Non-specific NF-κB inhibitor: SSZ | Competitively bind to the ATP pocket of the IKKα and IKKβ kinase ([38](#_ENREF_38)) | ↓ IL-6, IL-8, TNF-α and PGE_2_ production in LPS and γ-irradiation killed *E. coli* stimulated human fetal membrane explant model ([36](#_ENREF_36), [40](#_ENREF_40))  ↓ PTB rates in a mouse model of *E. coli* induced PTL ([41](#_ENREF_41)) | Approved for use in pregnancy to treat inflammatory disease (e.g. Crohn’s disease) ([39](#_ENREF_39))  No significant short term impact of these drugs on congenital abnormalities ([39](#_ENREF_39)) | Prolonged *in vitro* exposure (20 hours) resulted in increased levels of chorionic apoptosis and reduced cell viability ([40](#_ENREF_40)) |
| TLR-4 antagonists | Monoclonal anti-TLR-4 antibody bind to TLR-4 receptors and block LPS-TLR-4 interactions | ↓ TNF-α, IL-8 and PGE_2_ production in amniotic fluid in a nonhuman primate model of LPS induced PTB ([42](#_ENREF_42))  ↓ LPS induced PTB in a mouse model of PTL ([43](#_ENREF_43)) | Inhibits innate immune response at the very start without downstream inhibitory effects on constitutive NF-κB activity | Relatively early stage of research, further studies investigating adverse side effects required |
| TNF-α biologics | Anti-TNF-α antibodies and soluble TNF-α receptors that bind to and block TNF-α activity ([8](#_ENREF_8)) | ↓ PTB rates and pro-inflammatory IL-6 and IL-1β production in a mouse model of LPS induced PTB ([47](#_ENREF_47)) | Approved for use in pregnancy to treat inflammatory disease (e.g. Crohn’s disease) ([49](#_ENREF_49))  No significant short term impact of these drugs on congenital abnormalities ([51](#_ENREF_51)) | High levels in fetal circulation may increase risk of neonatal infection following maternal administration of antibody-based TNF-α biologics (e.g. infliximab) ([51](#_ENREF_51)) |

*Abbreviations: Adenosine Triphosphate (ATP); Interleukin (IL); Intrauterine infection (IUI); IκB kinase (IKK); Lipopolysaccharide (LPS); Matrix metalloproteinase (MMP); Mitogen-activated protein kinase (MAPK); N-acetyl cysteine (NAC); Nuclear factor κB (NF-κB); Preterm birth (PTB); Prostaglandin (PG); Sulfasalazine (SSZ); Toll-like receptor (TLR); Tumor necrosis factor (TNF)*
